# The Wear and Tear of Literary Life; or, the Last Days of Robert Southey
*The Life and Correspondence of Robert Southey. In six volumes. Edited by his son, the Rev. Charles Cuthbert Southey, M.A., Curate of Plumbland, Cumberland. London: Longman, Green, and Longman. 1851.


**Published:** 1852-01-01

**Authors:** 


### THE JOURNAL

OF

### PSYCHOLOGICAL MEDICINE

AND

### MENTAL PATHOLOGY.

---

JANUARY 1, 1852.

---

### ART. I.—THE WEAR AND TEAR OF LITERARY LIFE; OR, THE LAST DAYS OF ROBERT SOUTHEY.*

THE medical psychologist restricts his attention for the most part too exclusively to the study of the mind when, under eclipse, it may be observed passing through various phases of disease; but much instruction may also be derived from watching its course when, unaffected by the slightest aberration, it may be seen steadily traversing the ecliptic of its own glory. It has often occurred to us that it would be a striking, and indeed affecting spectacle, if we could only contemplate and contrast the same mind with itself at different periods of life; now exulting in the meridian of its brightness, and now sinking—"shorn of its beams"—perplexed, bewildered, and lost amidst the shadows which too often darken round the tomb of afflicted genius. "Walking in the fields, during the last summer," says an elegant writer, "I saw the sun going down in great glory, suddenly cut in two by a strip of dark cloud, which nevertheless showed itself, by the colour dimly shining through it, to be connected with that magnificent luminary; and while I stood, the vapour melted, and the sun reappeared in its large refulgence. My thoughts turned to the great lights that rule the intellectual day. I called to remembrance how the broad splendour of genius, as it rolls along the sky of life, from the morning until the evening, has its little intervals of shadow. The radiance of its manifestation is often broken. An inferior book or picture comes between the rising and the setting glory. A dark strip of cloud seems

* The Life and Correspondence of Robert Southey. In six volumes. Edited by his son, the Rev. Charles Cuthbert Southey, M.A., Curate of Plumbland, Cumberland. London: Longman, Green, and Longman. 1851.

22049

to cut the great light in the middle. It is a noble and comforting reflection that the gloom sometimes passes, the mind breaks forth again, and the poet or philosopher sinks behind the horizon of time, as he rose above it, in a full orb."* But alas! such was not the destiny of Robert Southey. The cloud that obscured his vigorous intellect never was dispelled—he did not, in his last moments, like his illustrious contemporary, Sir Walter Scott, recover even a transient gleam of consciousness. There was no clearing up of the mind before death, such as Aratæus has so well described, and such as we so often witness, especially in cases of mental disease. Utterly unconscious, worn out with physical suffering—his once athletic frame wasted to a shadow—this eminent historian, biographer, and poet, died at Keswick, on the 21st March, 1843.

The study of the human mind has hitherto been conducted, perhaps too exclusively, upon purely analytical principles. Locke, Reid, Dugald Stewart, and other psychologists, have aimed principally at classifying the different intellectual faculties; we would fain, however, contemplate the mind in its unital condition, and in so doing we cannot fail to be struck with that comprehensiveness which enables it to grasp and bring within its range every variety of knowledge. How comprehensive was the mind of Shakspeare, Milton, Dante, Bacon, Sir Isaac Newton! and it is this very comprehensiveness which constitutes one of the noblest attributes of the mind. "The striking peculiarity of Shakspeare's mind," says Hazlitt, "was its generic quality; its power of communication with all other minds—so that it contained a universe of thought and feeling in itself."† So also Channing, in his eloquent remarks on the character of Milton, observes, "Never was a more unconfined mind. The very splendour of his poetic fame has tended to obscure or conceal the extent of his mind, and the variety of its energies and attainments. Not only in the department of the imagination were his acquisitions vast—he travelled over the whole field of knowledge, so far as it had then been explored."‡ It may be well to analyse and compare the different intellectual faculties with each other—but if we would truly appreciate the greatness of the human mind, we must view synthetically all its different faculties in their collective vigour. It is not imagination alone, nor is it judgment alone, nor is it the exercise of reason alone, that will make a great Philosopher or a great Poet;

* Pleasure, Objects, and Advantages of Literature. A Discourse. By the Rev. Robert Aris Willmott, Incumbent of Bearwood, Berks. London: Bosworth. 1851. p. 53.

† Lectures on the English Poets. Delivered at the Surrey Institution. By William Hazlitt. London. 1818. p. 91.

‡ Remarks on the Character and Writings of John Milton. By W. E. Channing, LL.D. Boston. 1828.

but it is by the association and combination of all these different powers—one intellectual faculty now prompting the development of another, and now restraining its undue energy. We may decompose a sunbeam, but its elementary rays must be combined, in order that we may enjoy the blessing of sunlight. So is it with the mind. But this is not all. To estimate the character of men of genius, we must take into consideration the circumstances of the age in which they lived. There are, indeed, epochs in the intellectual as well as in the physical world—periods when the clouds of ignorance and superstition seem to break away under a blaze of intellectual light, which seems suddenly to illuminate the world. It is not the appearance of a single, but of many contemporaneous stars, which characterises these memorable eras. Such was the age of Queen Elizabeth, when Shakspeare, "not alone, but one of a race of giants,"—"in shape and gesture proudly eminent," moved in a constellation of bright luminaries, and drew after him a "third part of the heavens;"* such was the Augustan age of Anne, when the genius of Addison, Swift, Pope, Steele, and Prior, threw a zodiacal light across the literary firmament; and such also has been the first half of the present century, in which have flourished such men as Southey, Byron, Sir Walter Scott, Coleridge, Shelley, Wilson, Wordsworth, Campbell, Rogers, Keats, Crabbe, Barry Cornwall, Montgomery, and Tennyson. It is the same in the history of science—epochs as memorable at distant intervals appear, and different ages are characterized by such discoveries as were made by Copernicus, Galileo, Bacon, Newton, Sir Humphrey Davy; and, referring to the present period, by Sir David Brewster, Faraday, and Wheatstone. This sudden clustering together, as it were, of great minds, cannot easily be explained; but, without hazarding any speculation respecting the ultimate perfectibility of human nature, we certainly recognise in them evidence of mental progression in every successive age.

In studying the different phases through which the mind passes in a state of health, we cannot do better than study psychologically the biography of men of genius. It was a favourite observation of Wordsworth that "the life of a poet is written in his works." It is, so far as his moral nature is concerned, when he makes his hero impersonate his own sentiments and feelings. This Lord Byron did to a very great extent in Childe Harold. So likewise Wordsworth, in his "Prelude," recently published, in "The Excursion," and in almost all his minor poems, designedly embodied, as far as it was possible, the history of his own mind. It is a curious circumstance that Cowper, at the suggestion of a neighbouring clergyman, appears to have projected a work very similar to "The Excursion," which was to have been entitled "The Four Ages,"

* Hazlitt. Lectures. *Op. cit.*, p. 91.

wherein he was to have traced the history of the mind through Infancy, Youth, Manhood, and Old Age. "All who delight to accompany the genius of Cowper," says Hayley, "in animated flights of moral contemplation, will deeply regret that he was precluded by a variety of trouble from indulging his ardent imagination in a work that would have afforded him such ample scope for all the sweetness and all the sublimity of his spirit. His felicity of description, and his exquisite sensibility; his experience of life, and his sanctity of character, rendered him singularly fit and worthy to delineate the progress of nature in all the different stages of human existence."* We may incidentally remark that, between the poetry of Cowper and that of Wordsworth, there is in spirit and feeling a remarkable resemblance. Cowper was the poet of familiar life, and expressed simple truths in perfectly graceful and appropriate language—so did Wordsworth; Cowper excelled in verses of serious morality, and proposed to himself, as the main scope of his poetical labours, to establish "the service that a poet might be of to religion."† This, too, was throughout his life the object of Wordsworth. "Is not 'The Task,'" asks Burns, "a glorious poem? The religion of 'The Task,' bating a few scraps of Calvinistic divinity, is the religion of God and Nature—the religion that exalts and ennobles man." The mental-biography of the poet—if we may be allowed so to express it—may, it is true, be traced in such writings; but when the poet, in a higher mood of genius, divests himself of his own personality, and, inspiring life into ideal and self-consistent characters, exposes the secret workings of the human heart, we may derive instruction from the examples he places before us; but the author does not reveal himself to us. We learn nothing of the biography of Homer from studying the "Iliad" or the "Odyssey;" his name has become a myth; did such a person ever exist? Again. If we take up a volume of Shakspeare, we may dwell with admiration upon any particular tragedy or comedy, but we do not find "the life of the poet written in his works." Hartley Coleridge, who inherited much of the genius of his father, shrewdly observes, "Gladly as we would know more of our great dramatist, it is just as well so little is recorded;" upon which remark his biographer well observes, "If, indeed, the life of Shakspeare could be recorded; if we could be told how he thought, felt, and acted as an individual; how he bore himself under the pressure of the world, and with what mind he looked forward to another, the record might make us sadder, but would not make us wiser." Nevertheless, in a psychological point of view, such a record would be clearly interesting; for we hold that

* Life of William Cowper. By W. Hayley. 4 vols. 4to. London. 1804. Vol. ii. p. 172.

† Ibid., vol. ii. p. 444.

the biography of all men of genius should be studied in relation to the characteristics of their minds. We care little for Milton's domestic quarrels with his first wife; we take no interest in the frolics and escapades of Oliver Goldsmith; neither do we care to remember the particulars of the coffee-house brawl which perilled the life of Savage;—but every incident which throws light upon any particular feature of the mind, more especially those writings which reflect peculiar trains of thought and feeling, are invested with profound interest. When, therefore, we select the last days of Robert Southey for consideration, we do so strictly in a psychological sense. He was one of the most remarkable persons of his age. No man ever enjoyed a clearer intellect or a sounder judgment; no man was ever more indefatigable—his literary industry was unexampled; for many years of his life he was compelled, in his own words, to "drudge, drudge, drudge;" and it is by no means surprising that after over-taxing, or rather over-working, his brain for so many years, when domestic affliction fell heavily upon him, his naturally strong constitution should have given way, and that his mind should have sunk in eclipse below the horizon of existence.

Robert Southey was born August 12th, 1774. "The time of my birth," says he, playfully, in a letter to one of his friends, "was half-past eight in the morning, according to the family bible. According to my astrological friend Gilbert, it was a few minutes before the half-hour, in consequence of which I am to have a pain in my bowels when I am about thirty, and Jupiter is to be my deadly antagonist; but I may thank my stars for a gloomy capability of walking through desolation." His early love for books was manifested at eight years of age, when he tells us he read with delight Hoole's translation of the Gerusalemme Liberata, and Spenser's Faery Queen. "The first of my epic dreams," he tells us, "was created by Ariosto. I meant to graft a story upon the Orlando Furioso, not knowing how often this had been done by Italian and Spanish imitators. Arcadia was to have been the title and the scene; thither I intended to carry the Moors under Marsilius, after their overthrow in France, and there to have overthrown them again by a hero of my own, named Alphonso, who had caught the hippogriff. This must have been when I was between nine and ten, for some verses of it were written in my Phædrus."* It would be curious to trace the incidental circumstances which give a peculiar direction to certain mental faculties. In all children the faculty of imitation is very strong; and everything which pleases the infant mind is in itself suggestive of some corresponding train of thought. Not in the case of Southey only, but in many other instances, the perusal of one poem has suggested the composition of another, and to such accidental

* Life and Correspondence. Vol. i., p. 118.

circumstances we are indebted for one man becoming a great mathematician, another a great poet, another a great statesman. We do not, however, pretend to ascribe the peculiar direction which the mind of a man of genius takes exclusively to extrinsic causes. We must admit, despite all Locke has written to the contrary, the existence of certain intuitive principles, which, independent of education, give a natural bias and sometimes premature development to certain intellectual faculties. Lebrun at three years of age drew designs with chalk, and at twelve executed a portrait of his grandfather. Murillo filled the margin of his school books with drawings. Ferguson, the Scottish shepherd, while yet a boy, would, after the hours of his farm work were over, go into the fields, observe the stars, and calculate their distances by beads strung upon a thread, which he carefully laid down upon paper. Cowley, in the history of his own mind, compares the influence of boyish fancies to letters cut in the bark of a young tree, which grow and widen with it. "We are not surprised," says the Reverend R. A. Wilmot, "to hear from a school fellow of the Chancellor Somers, that he was a weakly boy, who always had a book in his hand, and never looked up at the play of his companions; to learn from his affectionate biographer that Hammond at Eton sought opportunities to steal away to say his prayers; to read that Tournefort forsook his college class that he might seek for plants in the neighbouring fields; or that Smeaton in petticoats was discovered on the top of his father's barn setting up a windmill which he had constructed. These early traits of character are such as we expect to find in the cultivated lawyer who turned the eyes of his age upon Milton; in the christian whose life was one varied strain of devout praise; in the naturalist who enriched science by his discoveries; and in the engineer who built the Eddystone lighthouse."* "Among the English poets," says Johnson, "Cowley, Milton, and Pope might be said to 'lisp in numbers,' and have given such early proofs, not only of powers of language, but of comprehension of things, as to more tardy minds seems scarcely credible."† While we recognise evidence of the existence of certain intuitive principles in the mind, we are not disposed to overlook the much which may be acquired by diligence, especially in acquiring the power of literary composition. La Fontaine had not the spirit of poetry awakened in him until he was in his twenty-second year, and then it was roused by his hearing an ode of Malherbes recited. Dryden gave no public testimony of his talents until he was twenty-seven, and Cowper did not become an author until he was fifty. To return to Southey. At the age of fourteen he was sent to Westminster, and at eighteen entered at Balliol College, Oxford. And now if we look to the

* Pleasures, Objects, and Advantages of Literature. *Op. cit.*, pp. 28, 29.
† Lives of English Poets. Cowley. Vol. vi., p. 3. Ed. London. 1824. 12 vols.

course of study which he adopted, and observe his habits of unremitting industry; if, more especially, we read the letters which he addressed to his different friends from college—for he was in early life a "lover of letter reading," we shall discover that it was entirely by excessive application and diligence that he laid the foundation of his future literary eminence. We do not recognise in the character of his mind any remarkable development, or manifestation of the faculty of imagination; and in after life we may observe that all his poems are, so to speak, of a composite character, that is to say, they are for the most part—[we refer to his larger poems, Joan of Arc, Madoc, Thalaba, the Curse of Kehama, and Roderic, and not to his ballads]—made up of historical facts or popular traditions felicitously versified. His command of language enabled him to rival Dryden and Collins in the cadences of his rhythm; but that creative faculty, and that plastic power of imagination, which abound so prodigally in the poetry of Shelley and Coleridge, were evidently wanting. He sat weaving poetry at the loom of art; it did not flow spontaneously as from an enchanted spring; nor was he ever carried away by those emotions which are the very soul of poetic feeling. Hence, when he was only twenty-five years of age, he writes to his friend William Taylor, of Norwich,* "Once I had a mimosa sensibility, but that has long been rooted out; five years ago I counteracted Rousseau by dieting upon Godwin and Epictetus; they did me some good, but time has done more. I have a dislike to all strong emotion, and avoid whatever could excite it; a book like Werter gives me now unmingled pain. In my own writings you may observe that I rather dwell upon what affects than what agitates."† Hence it is clear that Southey never would have succeeded as a dramatic poet. "It would be well," he observes, "if I *could* write tragedy—the true chrysopoetic vein. There are plans by me, and one opening scene, but I never had courage to proceed; and the sense of fear, and the disgust at trimming it to the taste of a green-room critic, have deterred me. Besides, if I know my own strength, it is in *narrative;* and dramatic parts introduced into narrative are widely different from the drama.‡ In another letter, he observes, "As to poetry, I have long abstained therefrom; old chronicles please me better."§ We conceive, therefore, that we are not doing injustice to the memory of Southey, when we affirm that he was not endowed, as Wordsworth expresses it, with "the vision and the faculty divine," albeit he had acquired "the accomplishment of verse." A somewhat remarkable illustration of this want of imagination is evinced

* Memoirs of the Life and Writings of the late William Taylor, of Norwich, containing his Correspondence of many years with the late Robert Southey. By J. W. Robberds. 2 vols. London: Murray. 1843. Vol. i., p. 262.

† Ibid., ii. p. 226. ‡ Ibid., vol. ii. p. 86. § Ibid., vol. i. p. 429.

in the opinion he gave of the "Ancient Mariner." "Coleridge's ballad," he observes, "of the Ancient Mariner, is, I think, the clumsiest attempt at German sublimity I ever saw."* The estimation, however, which men of genius, living in the same age, form of each other's productions, is often curiously hasty and unjust. Milton considered Dryden to be only a "man of rhyme," and highly esteemed the poetical abilities of Cowley; Gray underrated Akenside, and accused Collins of having "a bad ear," and "no choice at all of words and images." Yet have many of the odes of Collins, that to "Liberty," and that to "Mercy" especially, been not unjustly compared to some of the finest choruses in Euripides. Wharton pronounced judgment against the poetical diction of Milton, and there can be no doubt that the reviewers, upon the appearance of Wordsworth's "Excursion," did not appreciate, if they understood, the beauties and the philosophy of that noble poem. "Jeffrey," says Southey, "has, I hear, written what his admirers call a 'crushing' review of the 'Excursion;' he might as well beat himself upon Skiddaw, and fancy that he crushed the mountain."

When we proceed to analyse the peculiar traits or characteristics of Southey's mind, we cannot fail to observe, that as a poet his imagination—esteeming that as, in the highest sense, a creative or inventive faculty—was deficient or not developed in any remarkable degree. It is, however, justly observed by Dugald Stewart, that imagination should be regarded only as one of the many endowments of intellectual superiority. The steady exercise of reason and good sense in guarding and controlling this important faculty is essential, otherwise the imagination, once excited, becomes perfectly ungovernable, and produces something like a temporary insanity.† Even Coleridge, whose genius as a poet of great originality is, we believe, universally recognised, considered imagination should be counterbalanced, or held in subordination by other mental faculties. "The poet described in ideal perfection," he remarks, "brings the whole soul of man into activity, with the subordination of its faculties to each other, according to their relative worth and dignity. He diffuses a tone and spirit of unity that blends and, as it were, fuses each into each, by that synthetic and magical power to which we have exclusively appropriated the name of imagination. This power, first put in action by the will and understanding, and retained under their irremissive, though gentle and unnoticed control, (*laxis effertur habenis,*) reveals itself in the balance or reconciliation of opposite or discordant qualities; of sameness with difference; of the general with the concrete; the idea with the image; the individual with the representative; the

* Memoirs of the Life and Writings of the late William Taylor. Vol. i., p. 223.

† Philosophical Essays. By Dugald Stewart. Edinburgh. 1810. 4to. See On the culture of certain Intellectual Habits, p. 523.

sense of novelty and freshness with old and familiar objects; a more than usual state of emotion with more than usual order; judgment ever awake and steady self-possession; with enthusiasm or feeling profound or vehement; and while it blends and harmonizes the natural and the artificial, still subordinates art to nature, the manner to the matter, and our admiration of the poet to our sympathy with the poetry. Finally, good sense is the body of poetic genius, fancy its drapery, motion its life, and imagination the soul that is everywhere and in each, and forms all into one graceful and intelligent whole."* Elsewhere he observes, "No man was ever yet a great poet without at the same time being a great philosopher, for poetry is the blossom and fragrancy of all human knowledge, human thoughts, human passions, emotions, language."† It is evident that when imagination becomes unrestrained, and obtains a mastery over the other mental faculties; when it runs riot in the exuberance and prodigality of its own fictions, the most exalted conceptions become confused, and the imagery, like fragments of a broken mirror, may reflect brilliant, but nevertheless unconnected, hues. We have abundant evidence of this in the "Prometheus Unbound," and many other poems by Shelley, whose imaginative temperament was obviously intolerant of any mental discipline. We by no means, as we have just stated, wish to undervalue the merits of Southey as a poet; his "Madoc," "Joan of Arc," "Thalaba," "Curse of Kehama," and many of his minor poems, particularly some of his ballads and metrical tales, will doubtless continue to be admired; but they are all like pieces of "Mosaic work," evincing the triumph of art in poetical composition, rather than inspiration. In fact, Southey acquired, whether in prose or verse, a thorough command of the English language; and his versification, particularly in the poems of "Thalaba" and "Kehama," may fairly be compared with the "Absalom and Achitophel," the "Hind and the Panther," and some of Dryden's best poems. "I have read the first volume of 'Thalaba,'" says William Taylor, "and begin the next to-morrow. It contains a novel multitude of first-rate descriptive passages; it rivals in this respect Dryden's 'Alexander's Feast.'"

If the mind of Southey was not, as we affirm, characterized by any remarkable development of imagination,—viewing that faculty always in its inventive and creative sense,—he enjoyed other intellectual endowments practically even more valuable. His powers of judgment, comparison, and reasoning, were pre-eminently great, and enabled him, with the industry he possessed, to become the first historian, biographer, and essayist of his time. "It would be an extremely profitable thing,"

* Biographia Literaria; or, Biographical Sketches of my Life and Opinions. By S. T. Coleridge. London: Fenner. 1817. p. 12.

† Ibid., p. 21.

says the Rev. Sidney Smith, "to draw up a short and well-authenticated account of the habits of study of the most celebrated writers with whose style of literary industry we happen to be most acquainted. It would go far to destroy the absurd and pernicious association of genius with idleness, by showing that the greatest poets, orators, statesmen, and historians,—men of the most brilliant and imposing talents,—have actually laboured as hard as the makers of dictionaries and the arrangers of indexes, and that the most obvious reason why they have been superior to other men is, that they have taken more pains than other men."* There can be no doubt that exercising the intellectual faculties, like exercising the limbs of the body, will invigorate them, and that individual faculties will acquire thereby increased energy. The mind of Southey was ever active. "Literary exertion," said he, upon leaving college, "is almost as essential to me as meat and drink." This excessive application—this overtasking the powers of his nervous system—brought on, when he was twenty-six years of age, a malady painfully premonitory of the affliction which darkened his declining years. For the restitution of his health, it was found necessary for him to go to the south of Europe; and the fragmentary account we gather from his letters, of his own feelings and presentiments, clearly enough indicate that state of nervous irritability which we so frequently find predisposing to mental disease. Writing, Feb. 3, 1800, to William Taylor, he says—"I am seriously thinking of quitting England in search of health; either to wait till autumn, and then visit Lisbon, or to employ the summer in travelling through Vienna to Trieste. Something I must do, lest habits of sickness affect my mind as well as my body."† This lurking apprehension and dread of insanity we often find in persons who eventually succumb to that disease. It may be remembered that Dean Swift, walking with his friend Dr. Young, author of the "Night Thoughts," pointed to a noble elm, the uppermost branches of which were withered, and said prophetically, "I shall be like that tree—I shall die at the top." So also Sir Walter Scott, after his first apoplectic seizure, was haunted by the same dismal presentiment. "Such a shaking hands with death," said he, "is formidable. If I were worthy, I would pray God for a sudden death, and no interregnum between I cease to exercise reason and I cease to exist."‡ The medical psychologist will not fail to recognise in Southey's own account of his malady, symptoms which we constantly find to be precursory of cerebral affection. "My departure," he writes (Feb. 20, 1800), "will probably be

* Elementary Sketches of Moral Philosophy. Delivered at the Royal Institution in the years 1804, 1805, 1806. By the late Rev. Sidney Smith, M.A. London. 1850. p. 98.

† Memoir of the Life and Writings of William Taylor. Vol. i. p. 324.

‡ Memoirs of Sir Walter Scott. By J. Lockhart. Vol. vii. p. 252. Ed. 1837.

delayed until the autumn, and Lisbon the place of retreat. Go I must, or the worst consequences may result. Still I am ailing about the heart, and in spite of reasoning and probabilities, cannot but suspect, whenever its irregularities call my attention, that something is out of order about the mainspring. Connected with this at times, and at times recurring without it, are seizures in the head, like the terror that induces fainting —a rush through all my limbs, as if the stroke of annihilation were passing through me. This, then, seems decidedly nervous; but it must not be trifled with, for it threatens worse than the heart pain."*

Accompanied by his wife, Southey arrived at Lisbon on the 1st May, 1800, and the following July he thus describes his approaching convalescence in a letter to William Taylor, dated Cintra, July 5, 1800 :— "First of my health, the immediate object of this emigration. The effect of climate has been what I expected and wished. Night seizures I have none ; the irregularity of my heart is lessened, not removed ; I eat voraciously, and, above all, enjoy an everlasting sunshine of spirits. Something of this is assuredly owing to the total change of scenery and society, but the climate has been the great cause."† Here he continued unremittingly his literary pursuits. Independent of preparing "Thalaba" for the press, he busily employed himself collecting materials for an historical work on Portugal. "I am," he writes, "up to the ears in chronicles—a pleasant day's amusement, but battles and folios, and Moors and monarchs, *tease me terribly in my dreams.* I have just obtained access to the public manuscripts, and the records of the Inquisition tempt me—five folios the whole black catalogue." . . . . . "I obtain access through one of the censors of books here, an ex-German divine, who enlisted in the Catholic service, professing one faith with the same sincerity that he preached the other; a strong-headed, learned, and laborious man, curious enough to preserve his authoritative reviews of all that is permitted to be printed or sold in Portugal."‡ In the public library which was established by the ruin of the Jesuits, whose libraries were all brought to Lisbon, Southey found an invaluable *repertoire* of information respecting all that regards the peninsula, more especially church and monastic history. Here he collected materials for a history of Portugal, and many of those curious anecdotes connected with the early history of the Catholic church, which will be found in his "Vindiciæ Ecclesiæ Anglicanæ," the "Book of the Church," and other works. He had a keen perception of the ludicrous, and, as may be seen in the biography of Dr. Dove, in "The Doctor," any droll incident he could describe in the most minute and graphic style. His powers of observation were exceedingly acute, and throughout

* Memoir of William Taylor. *Op. cit.,* vol. i., p. 336.

† Ibid., vol. i. p. 348. ‡ Ibid., vol. i. p. 361

life he was as diligent a collector of *facts,* as any *virtuoso* of antique gems.

In the summer of 1801 he returned to England, and accepted an invitation to pass the autumn in Cumberland. "I am going," he writes (July 27, 1801), "to pass the autumn with Coleridge at Keswick, to work like a negro." He had some idea of becoming a barrister, but the study of the law was repulsive to him, and the atmosphere of London disagreed with him. "I grumble at nothing," he observes (Nov. 19, 1801), "but my compulsory residence in London, which I do loathe and abhor with all my moral and physical feelings." He therefore abandoned all intention of adopting the legal profession, and devoted himself entirely to literature. In the year 1794, when at Oxford, Southey became acquainted with Coleridge; both were young and inexperienced, and at this period, when the French revolution had spread its contagion throughout Europe, and old and young politicians crazed themselves with devising schemes for the reformation of society, these young men of ardent temperament, with some other companions,* united in a scheme of Socialism, which they designated Pantisocracy, and which they imagined would realize a state of society free from all the evils and turmoils which then agitated the world. "How do you go, my young friend?" asked Mr. Cottle, when one of these enterprising youths called upon him. "We shall freight a ship, and carry out with us ploughs and other implements of husbandry," was the answer. "When do you set sail?" "Very shortly; I expect my friends from the University, when all the preliminaries will be adjusted, and we shall joyfully cross the blue waves of the Atlantic." "But to freight a ship and sail out in the high style of gentlemen agriculturists will require funds; how do you manage this?" "We shall between us contribute what we can."† It is almost needless to add that this Utopian scheme fell to the ground, and can now only be referred to as an evidence of mistaken philanthropy. The amelioration of the social condition of mankind, by organizing society upon some new basis, so as to accomplish a state of ideal perfectibility—incompatible, we fear, with the frailty of humanity—has suggested theories of the same description to many sanguine minds. Unhappily, Shelley bewildered himself, and sadly misdirected his fine genius, in the mists of some such indefinite delusion. Wordsworth also set out in life as a republican, entertaining "too high an opinion of the human will, and too sanguine a hope of unlimited benefits to be conferred on society by the human

* Robert Lovell (a clever and accomplished young man, himself a poet), and George Barnet.

† Reminiscences of Samuel Taylor Coleridge and Robert Southey, By Joseph Cottle. London. 1847. pp. 7, 8.

intellect."* When experience and time temper down the imagination of such men, and mature the judgment, it is surely pardonable to find them change the theoretical opinions of their early life. An unalterable minister has been called an unalterable fool; and surely it may be permitted for minds highly gifted as these to correct their own erroneous impressions. To return to Southey. On the 14th November, 1795, under somewhat remarkable, or it might be said romantic circumstances, he was married at Redcliffe Church, Bristol, to Miss Edith Frecker, the sister of Mrs. Coleridge, and also Mrs. Robert Lovell. It had been arranged between them, on account of the unsettled state of Southey's worldly position, that their marriage should be kept secret. The day fixed for their union was that on which he was to set out for Lisbon. "Immediately after the ceremony," says his biographer, "they parted. My mother wore her wedding ring round her neck, and preserved her maiden name until the report of her marriage had spread abroad."†

In the August of 1803 they had the affliction of losing a favourite child, christened Edith, who died of hydrocephalus consequent upon teething. "My poor child," writes Southey, (August 24, 1813), "was buried yesterday, and we are quitting a place where everything reminds us of our loss. Poor Edith is almost heart-broken. I have gone through more suffering than I ever before experienced, for I was fond of her even to foolishness."‡ To relieve his distress by a change of scene, Coleridge, whom he had visited in 1800, persuaded Southey to remove to Keswick. Here Wordsworth, upon his return from Germany, in the spring of 1799, had already taken up his residence at Grasmere, and thus, from circumstances purely incidental, this brotherhood of poets became established in the same locality. At this period, or rather, a very few years afterwards, the star of Lord Byron rose in the ascendant. "Childe Harold," "The Corsair," "The Bride of Abydos," and "The Giaour," enchanted the public mind; and the only constellation that divided the literary hemisphere with Lord Byron was for some years the author of Waverley. It is true that Moore continued still pouring forth his charming Irish Melodies, which were in every society received with rapture; Shelley had shocked the world with the atheism of "Queen Mab," which, however, on examination, will be found expressive rather of pantheism in its widest sense ;§ John Keats had scarcely vindicated his claim to the poet's wreath; and Leigh Hunt and Hazlitt were in the zenith of their popularity—the one contributing to the current literature of the day poems, translations, and

* Memoirs of William Wordsworth, Poet Laureate, D.C.L. 2 vols. London: Moxon. 1851. Vol. i. p. 89.

† Life and Correspondence. Vol. i. p. 254.

‡ Memoirs of William Taylor. *Op. cit.*, vol. i. p. 468.

§ There is no doubt that Shelley was a thorough pantheist. What other principle essays remarkable for their exquisite taste and gracefulness; the other wielding the pen of Aristarchus with great originality of thought and force of language. Then arose through the length and breadth of the fashionable world a loud outcry against what was significantly called the "Lake School of Poetry;"—Wordsworth and Coleridge were ridiculed and caricatured in every conceivable shape, and Southey more especially, on account of changing his political opinions, was singled out as an object for attack. The criticisms which appeared at this period are now forgotten, or only referred to as a proof that public opinion will eventually award to every man of real merit retributive justice. There was, in point of fact, no confederation between these lake poets, who were accused of having in view the foundation of a particular school of poetry. "I am well pleased," says Southey (Jan. 11, 1803), referring to a criticism on "Thalaba," in the "Edinburgh Review," "to be abused with Coleridge and Wordsworth; yet it is odd enough my fellow-conspirator Wordsworth should be almost a stranger to me—a man with whom I have scarcely had any intercourse, not even a common acquaintanceship."* It is obvious that the mental characteristics of each of these poets, as expressed in their writings, are perfectly distinct and different, Coleridge delighting in the supernatural, in mysticism, and in the obscure sublime;—Wordsworth reflecting from the recesses of his heart feelings and thoughts which deified the most simple and familiar objects in nature;—while Southey, whose intellectual faculties were of another order, always dealt with palpable realities, weaving into matchless rhythm the historic tales and traditions which pleased his fancy. So little similarity existed intellectually between Wordsworth and Coleridge, that they could not throw their ideas or associate them together in the same channel, as is evinced by the curious history of that most remarkable poem, "The Ancient Mariner." It was agreed between these brother poets that they should defray the expense of a little tour by writing a poem for the "New Monthly Magazine," then edited by Dr. Aikin. "Accordingly," says Wordsworth, "we set off, and proceeded along the Quantock Hills towards Watchet; and in the course of this walk was planned the poem of 'The Ancient Mariner,'

could have suggested, even in the "pestiferous poem" of "Queen Mab" such lines as the following?—

"Spirit of Nature! here,  
In th' interminable wilderness  
Of worlds, at whose immensity  
Even soaring fancy staggers,—  
Here is thy fitting temple.  
Yet not the slightest leaf  
That quivers to the passing breeze  
Is less instinct with thee."

Memoirs of William Taylor. *Op. cit.*, vol. i. p. 440.

founded on a dream, as Mr. Coleridge said, of his friend Mr. Cruikshank. Much the greatest part of the story was Mr. Coleridge's invention, but certain parts I suggested; for example, some crime was to be committed which should bring upon the old Navigator, as Coleridge afterwards delighted to call him, the spectral persecution, as a consequence of that crime and his own wanderings. I had been reading in Shelvocke's Voyages, a day or two before, that while doubling Cape Horn, they frequently saw albatrosses in that latitude, the largest sort of sea-fowl, some extending their wings twelve or thirteen feet. 'Suppose,' said I, 'you represent him as having killed one of these birds on entering the South Sea, and that the tutelary spirits of these regions take upon them to avenge the crime.' The incident was thought fit for the purpose, and adopted accordingly. . . . We began the composition together on that, to me remarkable, evening. . . . As we endeavoured to proceed conjointly—I speak of that same evening—our respective manners proved so widely different, that it would have been quite presumptuous in me to do anything but separate from an undertaking upon which I could only have been a clog."* "The Ancient Mariner" thus originated. In his life of Cowley, Dr. Johnson describes what he is pleased to call a class of "Metaphysical Poets," and among them enumerates Donne, Derham, Suckling, Waller, Cowley, Cleiveland, and Milton, who, however, he adds, tried the "metaphysical style" only in his lines upon "Hobson the Carrier." The critical abilities of the great lexicographer so completely eclipsed his powers of imagination, that he estimated the value of poetry by measuring it only with a kind of metaphysical foot-rule; if the cadences did not scan to a nicety, or the images were not clearly defined and brought out into the strong light of realism, so as to leave nothing for the imagination to dwell upon, he was dissatisfied. And yet it is by no means necessary, nor even is it desirable, that either the poet or painter should supersede the faculty of imagination by entering too fully into descriptive details. Shakspeare, Goethe, and Sir Walter Scott understood this. Their female characters, *e. g.* Ophelia, Margaret in "Faust," Rebecca in "Ivanhoe," and we will add, "the lovely Lady Christabel," are marvellous and charming creations, because they leave an impression on the mind suggestive of more than we actually read of them. We remember on one occasion an artist of eminence called the attention of Sir Walter Scott to the miniature picture of a battle-field he was painting.—"Heh! man," said Sir Walter, "it's weel done—that lad is cleaving that chiel's helmet in right earnest, and that horse is dead eneugh—but that 's no the way to paint a battle. You should just raise a stour,† and let a glimmering

* Memoirs of Wordsworth. *Op. cit.*, vol. i. p. 108.

† "Stour" means a cloud of dust.

helmet be seen here, and a face there, and leave imagination to fill up the rest." This is truly applicable to that which we, instead of metaphysical, should designate psychological poetry. In the early part of the present century a spirit of German transcendentalism became transfused into our literature. Translations of the ballads and poems of Bürger, Goethe, Lessing, Wieland, Herder, Schiller, and the philosophy of Kant, became known, and certainly fascinated many highly gifted minds. We find William Taylor, whose name is indissolubly connected with English literature, translating, in amicable rivalry with Sir Walter Scott, several German ballads, and Sir Walter Scott bearing honourable testimony not only to the merits of his translation of Bürger's "Leonore," but to the influence it had upon his own career, which may be inferred from the following interesting anecdote:—One evening, Mrs. Barbauld, Dr. Aikin's sister, read this ballad to a circle of admiring friends at the house of Professor Dugald Stewart in Edinburgh. The impression which it made upon this highly cultivated and most intelligent assembly, who are described as having been "electrified by the tale," and the consequences which it immediately afterwards produced, have given to William Taylor's translation of "Leonore" considerable importance in the history of our literature. Not only did it serve to open the way for introducing into this country the works of the most eminent German poets, but it also supplied the spark by which the genius of one of the most remarkable and popular of our modern writers was first kindled. "Are you aware," said Mrs. Barbauld, in a letter addressed to William Taylor at a subsequent period, "that you made Walter Scott a poet? So he told me when, the other day, I had the gratification of meeting him. It was, he says, your ballad of 'Leonora,' and particularly the lines, 'Tramp, tramp along the land they speed,'* that inspired him. I do not wonder that any one able to appreciate that translation should speak thus of it."† This German style introduced a taste for romantic and supernatural tales, which Matthew Gregory Lewis, commonly known as Monk Lewis, carried in his "Tales of Wonder" and "Tales of Terror" to extreme. Nevertheless, he was a man of great original powers of mind, and possessed an exquisite ear for versification—which is sufficiently evinced in the popular ballads of "Durandarte" and "Alonzo the Brave." In Southey's "Madoc," "Roderick" and "Kehama," —and in Coleridge's "Ancient Mariner," "Christabel," "Tale of the Dark Ladie," we recognise the same Germanic spirit, which also is

* "Tramp, tramp along the land they speed;
Splash, splash along the sea.
Hurrah! the dead can ride apace;
Dost fear to ride with me?"

† Memoirs of William Taylor. *Op. cit.*, vol. i. p. 92. Lockhart's Life of Scott. Vol. i. p. 235.

diffused through many of Wordsworth's poems; indeed, were we asked for an illustration of what we mean by psychological poetry, we could scarcely select a better example than the following, from the "Lines composed a few miles above Tintern Abbey, on the Banks of the Wye." The poet describes the effect on his mind of the surrounding scenery:

> "These beauteous forms,
> Through a long absence, have not been to me
> As is a landscape to a blind man's eye;
> But oft, in lonely rooms, and 'mid the din
> Of towns and cities, I have owed to them,
> In hours of weariness, sensations sweet,
> *Felt in the blood and felt along the heart,*
> *And passing even into my purer mind*
> *With tranquil restoration:* feelings, too,
> Of unremembered pleasure; such, perhaps,
> As have no slight, or trivial influence
> On that best portion of a good man's life,
> His little nameless, unremembered acts
> Of kindness and of love. Nor less, I trust,
> To them I may have owed another gift,
> Of aspect more sublime: *that blessed mood,*
> *In which the burthen of the mystery*
> *Of all this unintelligible world*
> *Is lightened; that serene and blessed mood,*
> *In which the affections gently lead us on*
> *Until the breath of this corporeal frame,*
> *And even the motion of our human blood,*
> *Almost suspended, we are laid asleep*
> *In body, and become a living soul;*
> *While, with an eye made quiet by the power*
> *Of harmony and the deep power of joy,*
> *We see into the life of things.*"

Such poetry as this may truly be called psychological, the thoughts and feelings expressed being purely subjective; in other words, deriving their origin from the conceptions and emotions of the mind, independent of external objects. We find no poetry of this description in the writings of Southey; he dealt with the objective world rather than with the subjective. He was a great collector of facts, which his reasoning powers and judgment always disposed of to the best advantage; therefore is he one of the most instructive writers of the present period. These Lake poets, therefore, and we must include the highly-gifted professor of moral philosophy in Edinburgh, Professor Wilson, in their number, were not bound together by any confederate principle, but each possessed a very different order of mind.

The inveterate dislike to London which Southey entertained, and which probably arose from his health being there so much affected, induced him, after visiting Coleridge, to take up his residence at Keswick. In 1816 he writes, "London always affected my spirits; I breathe with difficulty, and positively hunger and thirst for fresh air." His friends Richard Duppa and William Taylor in this respect widely differed from him. Richard Duppa, when he visited his friends in Herefordshire, was always anxious to return to the metropolis, and boasted of being "town made," while William Taylor insisted that he never could perceive any beauty in mountain scenery.

"I never," he says, in answer to Southey, "could understand the merit of a mountain prospect: the eye walks on broken flints; the paths are too steep to ascend or descend; the rills too shallow to float a canoe; the hills too rugged for the plough; where there might be pasture glares a lake; cottages can be staked there, not a city." . . . . "How can you delight in mountain scenery? In the roads, every ascent is the toil of Sisyphus—every descent, the punishment of Vulcan: Barrenness, with her lichens, cowers on the mountain top, yawning among mists that irrigate in rain; the cottage of a man, like the eyrie of an eagle, is the home of a savage, subsisting by rapacity in stink and in intemperance; the village is but a coalition of pigstyes; the very cataract falls in vain—there are not customers enough for a water-mill. Give me the spot where victories have been won over the inutilities of nature by the efforts of human art. Where mind has moved the massy, everlasting rock, and arrayed it into convenient dwellings and stately palaces; into theatres, and cathedrals, and quays, and docks, and warehouses, wherein the primeval Troglodyte has learned to convoke the productions of the antipodes."*

"You undervalue lakes and mountains," retorts Southey; "they make me happier, and wiser, and better; and enable me to think and feel with a quicker and healthier intellect. Cities are as poisonous to genius and virtue, in their best sense, as to the flower of the valley or the oak of the forest."

It has been a subject of ingenious discussion, whether a residence in town or country be most desirable for men engaged in literature. In an article entitled the "Influence of Scenery on Poetic Character," which appeared in the old London Magazine,† (when that periodical was contributed to by Hazlitt,) Lamb, De Quincey, and other eminent contemporaries, contended that most of our great poets, with the exception of Shakespeare, were born and educated in the metropolis, and that a scanty field in its vicinity would be sufficient for men of genius, like Chaucer, Spenser, Milton, Dryden, Pope, or Cowper, to gather materials for their poetry. It has been also argued that Switzerland, rich in romantic scenery, has only produced a single poet. We do not acquiesce in this view. The choice between a town or country life will, for the most part, depend on the health, mental idiosyncracy, and habits of the individual; but as a general rule, beautiful scenery—whether sublime or picturesque

* Memoirs of William Taylor. *Op. cit.*, vol. i. pp. 413, 433.

† The London Magazine. September, 1821, p. 250. London: Taylor and Hessey.

—must be favourable to the development of poetic feeling. Hence, Milton, in his Tractate on Education, observes, "In those vernal seasons of the year, when the air is soft and pleasant, it were an *injury* and *sullenness* against nature not to go out and see her riches and partake with her rejoicings with heaven and earth." We certainly do not envy the disposition of the man who would prefer climbing to the top of Nôtre Dame to ascending a Swiss mountain, or who would choose to admire the gas-lights of a city, rather than the sublime expanse of the starry heavens. The effect of pure and fine scenic views must be in many states of mind obviously beneficial; and we can easily understand Southey exclaiming, upon his arrival at Keswick, "Would that you could see these lakes and mountains! how wonderful! how awful in their beauty! all the poet part of one will be fed and fostered here!" Accordingly, at Greta Hall, which commanded a magnificent prospect, Southey now took up his residence, and, with the exception of an occasional visit to London, Edinburgh, or Wales, the even tenour of his life was little varied. He had chosen literature as a profession, and dedicated himself entirely to its pursuit. His mode of life and habits were uniform, and had he not been a man naturally of strong bodily constitution, and had he not accustomed himself to taking very long walks among the mountains, his sedentary occupations, and constant exercise of the brain, would have much sooner impaired his health. One day with him passed like another day—

"My actions," he writes, "are as regular as those of St. Dunstan's quarter boys. Three pages of history after breakfast (equivalent to five in small quarto printing), then to transcribe and copy for the press, or to make my selections and biographies, or what else suits my humour, till dinner time; from dinner till tea I read, write letters, see the newspaper, and very often indulge in a siesta; for sleep agrees with me, and I have a good substantial theory to prove that it must, for as a man who walks much requires to sit down and rest himself, so does the brain, if it be the part most worked, require its repose. Well, after tea I go to poetry, and correct and re-write and copy till I am tired, and then turn to anything else till supper; and this is my life, which, if it be not a very merry one, is yet as happy as heart could wish. At least, I should think so, if I had not once been happier; and I do think so except when that recollection comes upon me, and then, when I cease to be cheerful, it is only to become contemplative—to feel at times a wish that I was in that state of existence which passes not away; and this always ends in a new impulse to proceed, that I may leave some durable monument and efficient good behind me."

"His course of life," says his son, "was the most regular and simple possible: he varied but little from the sketch he gave of it in 1806, which we have here given. When it is said that breakfast was nine after a little reading, dinner at four, tea at six, supper at half-past nine, and the intervals filled up with reading and writing, except that he regularly walked between two and four, and took a short sleep before tea, the outline of his day, during those long seasons when he was out of work, will have been given. After supper, when the business of the day seemed to be over, though he generally took a book, he remained with his family and was open to conversation, to amuse or be amused. It was at such times that the most pleasant fireside chatting, and the most interesting stories, came forth, and it was indeed at such time, though long before my day, that 'the Doctor' was originated, as may be seen by the beginning of that work, and the preface to the new edition. Notwithstanding the very mention of 'my glass of punch' —the one temperate never-exceeded glass—may be a stumbling-block to some of my readers, I am constrained, by the very love of the perfect picture which the first lines of 'the Doctor' convey of the conclusion of his evening, to transcribe them in this place. It was written but for a few; otherwise 'the Doctor' would have been no secret at all; but those few who knew him in his home will see his very look while they re-peruse it, and will recal the well-known sound of his voice:—" I was in the fourth night of the story of the Doctor and his Horse, and had broken it off, not like Scheherazade, because it was time to get up, but because it was time to go to bed. It was at thirty-five minutes after ten o'clock, in the year of our Lord 1813. I finished my glass of punch, tinkled the spoon against its side, as if making music to my own meditations, and having fixed my eyes upon the Bhow Begum, who was sitting at the head of her own table, I said, 'It ought to be written in a book.' "

Passages of this description show that Southey, like Sterne, was a minute observer of facts; many parts of the Doctor, indeed, are written very much in the style and manner of "Tristram Shandy."

The life of Southey, in the midst of such occupations, was not unchequered with anxiety; he had now an increasing family, the support of whom depended entirely upon his literary exertions; and frequently the painful idea would cross his mind, that if anything happened to him they would be left unprovided for. There are some minds which receive impulse from the sense of pecuniary emergency; otherwise, Johnson never would have written "Rasselas," or Goldsmith "The Vicar of Wakefield;" but there are other minds so constituted, that excessive anxiety will destroy all energy, and, as it were, paralyse the intellectual faculties. This was keenly felt by Coleridge. In what he termed "An affectionate Exhortation to those who in early Life feel themselves disposed to become Authors," he makes the following striking appeal:—

"With no other privilege than that of sympathy and sincere good wishes, I would address an affectionate exhortation to the youthful Literati, grounded on my own experience. It will be but short, for the beginning, middle, and end converge into one charge. NEVER PURSUE LITERATURE AS *a* TRADE. With the exception of one extraordinary man (*query* Southey ?), I have never known an individual—least of all, an individual genius—healthy or happy *without* a profession; *i. e.*, some regular employment which does not depend on the will of the moment, and which can be carried on so far mechanically, that an average *quantum* only of health, spirits, and intellectual exertion, are requisite to its faithful discharge. Three hours of leisure, unalloyed by an alien anxiety, and looked forward to with delight as a change and recreation, will suffice to realise in literature a larger product of what is truly genial, than weeks of compulsion. Money and immediate reputation form only an arbitrary and accidental end of literary labour. The hope of increasing them by any given exertion will often prove a stimulant to industry; but the *necessity* of acquiring them will, in all works of genius, convert the stimulant into a narcotic. Motives, by excess, reverse their very nature, and, instead of exciting, stun and stupify the mind."*

But this was not the case with Southey. He occupied himself in writing history, biography, poetry, political and moral essays, critical reviews, and carried on at the same time a very extensive epistolary correspondence. His life is in fact written in his letters; and these are so numerous, and dispersed in so many hands, that a considerable number of them remain yet unpublished. Many have been submitted to our inspection. They are written in a small, clear, and legible manner, with very few corrections or interlineations; and evince those mental characteristics we have above described. The following letter, describing his visit to Edinburgh, and his impressions respecting Sir Walter Scott and Jeffrey, will be read with interest:—

"Dear ———: Our succession of visitors is over—the summer birds have all taken flight. The Islanders are gone, the general gone, and our inn-door circle also contracted. Harry and Miss Barker have left us; the season for reviewing is begun, and I have put on my winter clothes and commenced my hybernation. My Scotch excursion with Elmsley was a pleasant one. We saw Melrose on our way; if not the most picturesque mass, certainly the finest architectural one in the whole island. We stayed three days with Sir Walter Scott, at his house on the bank of the Tweed. One morning was given to salmon-spearing, with a heavy trident about twelve feet long. I had to manage one end of a flat-bottomed, crazy boat, as she floated sideways down a crazy stream, and to keep her even, and prevent her striking against the rocks, and so upsetting. I did my part well, and having no evil designs upon the salmon, came home quite innocent, and sufficiently instructed in a very singular savage sport. Scott is a pleasant man, of open and friendly manners, so full of topographical anecdotes, that, having seen him, you would be perfectly well satisfied how well history may be preserved by tradition. We saw much classic ground, besides the Tweed. The Yarrow, with Newark castle, Branksome, overlooking the Teviot, and Johnny Armstrong's stronghold on the Esk. At Edin-

* Biographia Literaria. *Op. cit.*, i. p. 222.

burgh, Jeffrey was invited to meet me. Before he could venture to do this, he sent me his reviewal of 'Madoc,' then printed, but not published. A man who has been reviewed above fifty times, which is my case, is hardened to such things. Besides, by God's blessing, such praise or such censure as can be bespoken for five or ten guineas a sheet can neither help nor harm me now. They who fling dust at me will only dirt their own hands, for I am out of reach. So Jeffrey and I met constantly, and live very good friends. In fact, I am not very irascible; and if I had been so, found him too little to be angry with —a man of ready wit, no taste, and so little knowledge, that it would have been scarcely inaccurate to have said none. . . . . He has been to the Lakes, and supped with me. Of all the Scotch reviewers that have come in my way—and, with the exception of Sydney Smith, I have seen all of any celebrity—I think little, perhaps too little; but having lived with Coleridge, and Wordsworth, and William Taylor, it is impossible not to perceive that these Scotchmen are very feeble indeed. . . . . I have seen the Monthly Review of 'Madoc.' Some wretched man, who either has been reviewed by me with deserved severity, or fancies so, has been permitted to vent his spleen there, which he has done very clumsily. It is stupid and blunt ill-nature—a bluebottle fly, wriggling his tail, and fancying he has a sting in it. Edinburgh is the finest city I have ever seen. Having no new coat since I was in London, and no new hat, except a seven-shilling white one of felt, it was judged proper by Edith that I should beautify my appearance in Scotland, and also adorn myself with new boots and new pantaloons; but when I saw them, and contemplated the very respectable figure I already made, considering the vanity of externals—and moreover remembered, that as learning was more valuable than house and lands, it must be much better than new clothes, I laid out all my money in books, and have, in consequence, the pleasure of laughing at the manœuvre and reading the books. After this year, at all events, I have done with reviewing, and heartily glad shall I be to leave off the trade."

Although Southey had been reviewed above fifty times, as he informs us, and was "hardened to such things," it is clear, from the bitterness with which he alludes to the criticism on "Madoc" in the "Monthly Review," that he was still vulnerable; nay, notwithstanding his many very admirable mental and moral qualities, he frequently formed very hasty judgments, and when offended could not divest himself of personal prejudices—witness the disparaging tone in which he always refers to Godwin. There can be no doubt that Southey felt hurt at the criticisms upon his poems which appeared in the "Edinburgh Review," and formed an unfavourable opinion, not only of the "Review" itself, but of its contributors. In writing to Coleridge, in 1803, he says—"The 'Edinburgh Review' will not keep its ground; it consists of pamphlets, instead of critical accounts." This was clearly a false prophecy. We cannot also help remarking, that when Southey pronounced so decided a judgment against his Scotch contemporaries, he had been in Edinburgh only for a very short period, and had not an opportunity of entering a society then adorned with such men as Sydney Smith, Adam Ferguson, John Playfair, Dugald Stewart, Lord Kinnedder (Erskine), Henry Mackenzie, Francis and Leonard Horner, and many other men equally distinguished. The literary circle in Edinburgh was at this very period particularly brilliant. In all the letters to his friends which he wrote, giving an account of his visit to Scotland, many of which have been already published, he describes Jeffrey in the same language; but the injustice of such an opinion needs no comment. Very different was the impression of his friend William Taylor, who, in speaking of Jeffrey, observes—

"It is not with his politics I am in love, but with his comprehensive knowledge, with his brilliant and definite expression, and with his subtle argumentative power. I have not yet seen the 'Quarterly Review;' it is said to rival that of Jeffrey, but *I shall be surprised if there is literary strength enough in any other combination to teach so many good opinions as the Edinburgh Review.*"*

The account Southey gives of spending his money in books rather than in clothes is amusing. Books, indeed, were not only his delight—the purchase and possession of them became with him a passion. His house, from the roof to the basement, was fitted up as a library; every room and passage, every closet and cranny, were made available for holding books.

"His own sitting-room, which was the largest in the house," his son tells us, "was fitted up with the handsomest of them, arranged with much taste, according to his own fashion, with due regard to size, colour, and condition; and he used to contemplate these, his carefully accumulated and much-prized treasures, with even more pleasure and pride than the greatest connoisseur his finest specimens of old masters. His Spanish and Portuguese collection was the most highly-prized portion of his library, and comprised a considerable number of valuable MSS., which had been copied out of private and convent libraries. Many of these old books being on vellum or parchment bindings, he took pains to render ornamental to portions of his shelves. His brother Thomas was skilful in caligraphy, and by his assistance their backs were painted with some bright colour, and upon it the title placed lengthwise in large gold letters, of the old English type. Another fancy of his was to have all those books of lesser value, which had become ragged and dirty, covered, or rather bound, in coloured cotton print, for the sake of making them clean and respectable in their appearance, it being impossible to afford the cost of having so many put into better bindings. Of this task his daughters, aided by any female friends who might be staying with them, were the performers; and not fewer than from

* Memoirs of William Taylor. *Op. cit.*, vol. ii. p. 272.

1200 to 1400 volumes were so bound by them at different times, filling completely one room, which he designated the COTTONIAN LIBRARY. With this work he was much interested and amused, as the ladies would often suit the pattern to the contents, clothing a quaker work or book of sermons in sober drab, poetry in some flowery design, and sometimes contriving a sly piece of satire at the contents of some well-known author, by their choice of its covering."*

Here, like a Benedictine monk in a convent, as Wordsworth observed, he pursued, winter and summer, his literary avocations.

In one of the letters before us he observes—

"We go on well. I never go beyond the premises, though our weather has been delightful, more so than ever winter was remembered here. The snow has never covered the valley half-an-hour during the whole winter. We live as completely without society as if we were in Kamschatka; but summer is coming on, and then there will be too much of it. I get on steadily with my opus magus, the history, and only wish that I were rich enough to have an amanuensis at hand, and to buy all the books that would be useful to me."

In speaking of his brother's return home, he thus affectionately expresses himself—

"My brother Tom arrived yesterday from sea, and my spirits have not recovered their usual temperate tone, for it dispirits me to see him looking prematurely old; to think that in fourteen years he has been only nine months ashore, and that we three brothers, who are now in one house, have never been together till now during the whole of that period, and may very possibly separate in a few weeks, and may never meet together again. Family ties, if they are good for anything, grow stronger as we grow older, and as fewer are left us. We then feel how different they are from other friendships, be these friendships ever so sincere. I will never breed up a child to the navy or army, nor send one to the East Indies. It is very well for birds, whose love is only instinct, to be turned adrift as soon as they leave the nest; but it is an evil thing for a family to be scattered."

In many passages he amusingly refers to Coleridge. In one he says—

"Coleridge is appointed confidential secretary to Sir A. Ball, at Malta, and is going in the spring up the Black Sea to purchase corn for government. I should as soon think of setting him to cut a corn for me, though he will do the business as well, and more honestly, than most people."

In another letter—

"Of Coleridge we know nothing. He wrote to me that he would write again, if he could, that same evening. This was more than three weeks ago. Wordsworth has just had a few lines to say he is gone to

* Life and Correspondence. Vol. vi., p. 8.

Margate. In anybody else, this would be very odd; but comets and Coleridge baffle all calculation."

His kind feelings towards Wordsworth, and his sympathy with him upon the melancholy occasion of his brother Captain Wordsworth's death, who was lost in the wreck of the vessel he commanded,* he thus tenderly expresses :—

"My time has been taken up on a very distressing occasion. I have been over to poor Wordsworth and his sister, who are almost heart-broken by the dreadful fate of their favourite brother, in the Abergavenny. Nothing which did not immediately come home to myself ever affected me so deeply. I am going over again in two or three days, and much of my time will be thus employed until they, in some degree, get the better of their affliction."

We intentionally pass over Southey's appointment as secretary to Mr. Corry, the Chancellor of the Exchequer for Ireland, which situation he held only for a short period; also the circumstances connected with his succeeding Henry James Pye as poet laureate, our object being to dwell upon those habits of life, and manifestations of thought and feeling, which, psychologically considered, reveal to us the peculiar characteristics of his mind. His personal appearance and manner indicated a man of great nervous excitability—

"His forehead," we are told, "was very broad; his height, five feet eleven inches; his complexion rather dark; the eyebrows large and arched; the eye well-shaped and dark brown; the mouth somewhat prominent, muscular, and very variously expressive; the chin small in proportion to the upper features of the face. The characteristics of his manner, as of his appearance, were lightness and strength; an easy and happy composure was the accustomed mood, with much mobility at the same time, so that he could be readily excited into any degree of animation in discourse, speaking, if the subject moved him much, with extraordinary fire and force, though always in light laconic sentences. When so moved, the fingers of his right hand often rested against his mouth, and quivered through nervous susceptibility."†

The medical psychologist will not fail to recognise in this description evidence of his possessing a highly nervous temperament, such as rendered him quick in feeling, and liable to be affected deeply by domestic affliction. By adopting very regular habits, by taking a great deal of pedestrian exercise, and by pursuing his studies in a very systematic manner, he counteracted for many years an obvious proclivity to ner-

* The *Earl of Abergavenny* East-Indiaman, commanded by Captain Wordsworth, was wrecked on the shambles of the Bill of Portland, on Tuesday, February 5, 1805. A very affecting account of the event, and the distress of Wordsworth's family, will be found in the "Memoirs of Wordsworth, by Christopher Wordsworth, D.D." Vol. i. chap. xxii. p. 181. This is one of the most melancholy chapters in modern biography.

† Ibid., vol. vi. p. 281.

vous disease. The way in which he, in reading, arranged for information and reference the contents of a book, may be cited in illustration of his methodical habits:

"He was," says his biographer, "as rapid a reader as could be perceived, having the power of perceiving, by a glance down the page, whether it contained anything he was likely to make use of: a slip of paper lay on his desk, and was used as a marker, and with a slight pencilled S he would note the passage, put a reference on the paper, with some brief note of the subject, which he would transfer to his note-book, and in the course of a few hours he had classified and arranged everything in the work which it was likely he would ever want. It was thus, with a remarkable memory (not so much for the facts or passages themselves, but for their existence, and the authors that contained them), and with this kind of index both to it and them, that he had at hand a command of materials which have been truly said to be unequalled. Many of the choicest passages he would transcribe himself, or employ one of his family to transcribe for him; and these are the extracts which form his 'Common-place.' There can be no doubt that persons who accustom themselves to taking notes are apt to rely upon referring to them, and, therefore, do not take the same pains in charging the memory with them, and, from not being exercised, this intellectual faculty becomes impaired. This was felt by Southey, who, in conversation with Dr. Shelton Mackenzie upon a question touching dates, remarked—'I could as soon fly as recollect these dates. I have trusted so little to memory, that memory will do little for me when I press her. I have a habit of making notes of what I should treasure up in my mind, and the act of writing seems to discharge it from my mind to the paper.'"

As life advanced, the nervous excitability of Southey's temperament obviously increased, and we find him in the prime of life (ætat. 45), and in the zenith of his fame, dwelling with painful anxiety on the aspect of the political world, and giving way to feelings of morbid apprehensions as to future events. The following letter, addressed to his friend Grosvenor Bedford, dated Keswick, Dec. 5, 1818, reveals to us a state of mind upon which we, as mental pathologists, would have pronounced a very unfavourable prognosis:—

"My dear Grosvenor," he writes, "it is between ourselves a matter of surprise to me that this bodily machine of mine should have continued its operations with so few derangements, knowing, as I well do, its excessive susceptibility to many deranging causes. The nitrous oxide" (which Sir Humphry Davy had then just discovered) "approaches nearer to the notion of a *neurometer* than anything which perhaps could be devised; and I was acted upon by a far smaller dose than any one person upon whom it had ever been tried, when I was in the habit of taking it. If I did not vary my pursuits, and carry on many works of a totally different kind at once, I should soon be incapable of proceeding with any, *so surely does it disturb my sleep and*

*affect my dreams if I dwell upon one with any continuous attention. The truth is, that though some persons, whose knowledge of me is scarcely skin deep, suppose I have no nerves because I have great control as far as regards the surface; if it were not for great self-management and what may be called a strictly intellectual regimen, I should soon be in a deplorable state of what is called nervous disease, and this would have been the case any time during the last twenty years.* . . . I want now to provide against that inability which may any day or any moment overtake me. You are not mistaken in thinking that the last three years have considerably changed me; *the outside remains pretty much the same, but it is far otherwise within.* If hitherto the day has been sufficient for the labour, as well as the labour for the day, I now feel that it cannot always, and possibly may not long be so. Were I dead, there would be a provision for my family, which, though not such as I yet hope to make it, would yet be a respectable one. But if I were unable to work, half my ways and means would be instantly cut off, and the whole of them are needed. *Such thoughts did not use to visit me. My spirits retain their strength, but they have lost their buoyancy, and that for ever.* I should be better for travelling, but that is not in my power. At present the press fetters me, and if it did not, I could not afford to be spending money when I ought to be earning it. But I shall work the harder to enable me so to do."*

Twenty years previous to this, as we have above seen, the same gloomy foreboding, the same ominous presentiment, crossed his mind; is there not something prophetic in such spiritual forewarning? May not the apprehension and dread of the calamity be in itself an exciting cause of it? To work indeed he set with increased earnestness, and when, the following year, he projected a visit to his friend in London, he writes to him thus before commencing the journey—

"I have to finish 'Wesley,' which will be done in five weeks, taking it coolly and quietly. I have to finish the review of 'Marlborough,' which will require three weeks. One of them is my morning, the other my evening's work. If I am satisfied about the payment for my last paper, I shall recast the article upon the new churches, and perhaps prepare one other also, in order to be beforehand with my ways and means for the spring of next summer. The 'Tale of Paraguay' has proceeded more slowly than tortoise, sloth, or snail; I must finish it for publication in the ensuing year, or I shall not be able to keep my head above water."

Such is the life of a literary man in active employment; "Needle and stitch, needle and stitch," as the poor sempstress sings in Hood's pathetic "Song of the Shirt," he may truly re-echo. "Look," cries Carlyle, "to the biography of men of letters; with the exception of the 'Newgate Calendar,' it is the most sickening chapter in the history of man."

* Life and Correspondence, *loc. cit.*

We have only briefly alluded to Southey's marriage; he was, at the period to which we now allude, the father of several children; his wife keenly participated in all his anxieties, and she and her daughters might often be seen, sitting at the same table, copying out passages from books which he had marked to be extracted, all uniting cheerfully in assisting in those great literary undertakings which were the prop and honour of their house. The health of Mrs. Southey was delicate; the precarious fortune of her children, and probably Southey's own gloomy anticipations, preyed upon her mind. Her despondency increased; she became gradually more and more restless and unsettled; until at length a total loss of appetite and want of sleep excited the most serious alarm. The usual precursory symptoms of this form of mental disease went on increasing until it eventually became apparent to her afflicted family that she was "no longer herself." "It is, perhaps," observes the Rev. Cuthbert Southey, "rash to endeavour to search into the causes of these mysterious visitations of providence; but it may, I think, fairly be alleged, that an almost life-long anxiety about the uncertain and highly precarious nature of my father's income, added to a naturally nervous constitution, had laid the foundation of this mental disease; and my father himself now felt and acknowledged that Keswick had proved, especially of later years, far too unquiet a residence for her weakened spirits." With deep reluctance, but yielding to the imperative necessity of the case, her removal to an asylum was determined upon, as "affording the best, if not the only, hope of restoration; accordingly, she was removed to the Retreat at York." It is impossible to describe the distress of Southey; and it would almost be impertinent to attempt doing so in any other than his own language. In writing to his friend Grosvenor he says :—

"After what Henry Taylor has imparted to you, you will not be surprised at learning that I have been parted from my wife by something worse than death. Forty years has she been the life of my life, and I have left her this day in a lunatic asylum. God, who has visited me with this affliction, has given me strength to bear it, and will, I *know*, support me to the end, whatever that may be. Our faithful Betty is left with her; all that can be done, by the kindest treatment and the greatest skill, we are sure of at the Retreat. I do not expect more than that she may be brought into a state which will render her perfectly manageable at home. More is certainly possible, but not to be expected, and scarcely to be hoped. To-morrow I return to my poor children. There is this great comfort—that the disease is not hereditary, her family having, within all memory, been entirely free from it. I have much to be thankful for under this visitation. For the first time in my life, I am so far beforehand with the world, that my means are provided for the whole of next year, and that I can meet this additional expenditure, considerable in itself, without any difficulty.

Another thing I am thankful for is, that the stroke did not fall upon me when the printers were expecting the close of my naval volume, or the 'Memoir of Dr. Watts.' To interrupt a periodical publication is a grievous loss to the publishers, or, at least, a very serious inconvenience. Some old author says, "Remember, under any affliction, that time is short; and that though your cross may be heavy you have not far to bear it.' I have often thought of those striking words."

This melancholy letter was dated, York, Thursday night, October 2, 1834; and the morning following, addressing, in the same mournful tone, his friend Taylor he says :—

"Yesterday, I deposited my dear wife in the Retreat for lunatics, near the city, and to-day I visited her there. To-morrow I return home to enter upon a new course of life. Recovery is possible; but I do not attempt to deceive myself by thinking that it is likely. It is very probable that she may be brought into a state which will no longer require restraint. In that case I shall engage a proper attendant from this place, bring her home, appropriate two rooms to her use, and watch over her, to give her all the comforts of which she may be capable, till death do us part. The call upon me for exertion has been such that, by God's help, I have hitherto felt no weakness. That this is a far greater calamity than death would have been, I well know. But I perceive that it can be better borne at first, because there is a possibility of restoration, and, however feeble, a hope. Therefore, that collapse is not to be apprehended, which the circumstances of a mortal sickness, and death, and burial, call forth in the survivor, is at an end. Mine is a strong heart. I will not say, that the last week has been the most trying of my life; but I will say, that the heart which can bear it, can bear anything. It is remarkable, that the very last thing I wrote, before this affliction burst upon me, was upon resignation."

Upon his return to Keswick, he was surprised by a letter from his friend Taylor, offering to receive his daughters in his house :—

"Thank you, most heartily," he answers, "for your offer; but, at present, it is better that I should be alone, and that the girls should be left to themselves with Miss Hutchinson. For me this is best, because nothing is so painful as a reaction of your own thoughts after you have been for awhile drawn away from them, if this be attempted too soon. When I can enjoy your company, I shall be most thankful for it; and as you know I shall not give myself to melancholy, you need not apprehend any ill consequences from my being alone."

Truly, observes his affectionate biographer, this was an awful separation between those who had been so long, so truly united;—to this, death had been a light evil, for when are we so near as then,—

'Tis but the falling of a leaf,
The breaking of a shell,
The rending of a veil.

But what a gulf is there "fixed" between the reasoning and the unreasoning mind. While the affliction was yet recent—and there was room even to hope against hope, Southey contended manfully with the grief which nevertheless was inwardly undermining the stability of his own mind.

"He kept up, indeed," continues his son, "wonderfully, and a common observer would have remarked but little change in him, except that he was unusually silent; but to his family the change was great indeed; yet he bore the trial patiently and nobly; and when, in the following spring, it was found that the poor sufferer was likely to be better under his own roof, and the period of suspense and doubt, and alternate hope and fear, had passed away, it was marvellous how much of the old elasticity remained, and how, though no longer happy, he could be contented and cheerful, and take pleasure in the pleasures of others."

About three weeks after his return home, he writes:—

"This morning's letter is decidedly favourable, and I feel its effects. Hitherto, I have not recovered my natural sleep at night; plenty of exercise and quiet employment fail of their wonted effect in producing, because, in darkness and solitude, uncomfortable thoughts prevent sleep for awhile, and then trouble it. I should not be better for society, nor for leaving home. There is nothing to be done but to pursue the same course of self-management, live in as much hope as it may be reasonable to encourage, and above all, to bear always in mind that we have entered upon the last of our seven stages. In a very few years, what may have befallen us in the course of these years may be of some interest to any one who may write my life; but it will be of no consequence to us, whose lot, doubtful as it is for the short remaining portion of our time, is, I trust, fixed for eternity."

A little later, to another friend he writes:—

"I am beginning to sleep better the last few days, and I do everything that is likely to keep myself in bodily and mental health—walking daily in all weathers, never overtasking myself, or forcing myself to a distasteful employment, yet never remaining idle. But my spirits would assuredly give way were it not for a constant reference to another world, and a patient hope of God's mercy in this."

The reputation which Southey had achieved—the marked influence which his polemical writings in defence of the established church and the prerogatives of the crown had at various times had on public opinion; and the numerous friends by whom he was admired and esteemed, necessarily caused the subject of his affliction to be a topic of much conversation. His society, when he visited London, which he only did occasionally, had been sought by persons of the highest rank and consideration; in illustration of which we may cite the following interesting little anecdote of her present majesty:—

"Upon one occasion he received an invitation to dine with the Duchess of Kent, at Kensington Palace, and at the conclusion of the repast, before the ladies had retired, the young Princess Victoria came up to him, and curtseying gracefully, said to him very prettily, 'Mr. Southey, I thank you for the pleasure I have received in reading your 'Life of Lord Nelson.'"*

We are not, therefore, surprised to find that Sir Robert Peel—that munificent patron of literature and the fine arts—when prime minister, wished to confer some marked honour on a man so distinguished. He was probably ignorant of his domestic circumstances :—

"One morning," says our biographer, "shortly after the letter had arrived, my father called me into his study, and said, 'You will be surprised to hear that Sir Robert Peel has recommended me to the King for the distinction of a baronetcy, and you will probably feel some disappointment when I tell you that I shall not accept it, and this more on your account than my own. I think, however, you will be satisfied I have done so for good and wise reasons.' He then read to his son Sir Robert Peel's letter, stating that he 'had advised the King to adorn the distinction of baronetage with a name the most eminent in literature, and which had claims to respect and honour which literature only can confer;' and Sir Robert added that 'the King most cordially approved of his proposal.'"

This official letter was accompanied by another, marked "private," couched in the warmest and most friendly terms. He then read to his son the answer he had written, declining the honour; and as it is essential, in considering the fluctuations of bodily and mental health, to take into consideration those extrinsic circumstances which operate as exciting causes, we have thought it right to give this letter at length. It describes Southey's circumstances at the time so very fully, and is in some parts so affectingly written, that it cannot fail to be read with interest. He says :—

"Dear Sir,—No communications have ever surprised me so much as those which I have this day the honour of receiving from you. I may truly say also, that none have ever gratified me more, though they make me feel how difficult it is to serve any one who is out of the way of fortune. An unreserved statement of my condition will be the fittest and most respectful reply. I have a pension of 200*l.*, conferred upon me through the good office of my old friend and benefactor, Charles W. Wynn, when Lord Grenville went out of office; and I have the Laureateship. The salary of the latter was immediately appropriated, as far as it went, to a life insurance for 3000*l.* This, with an earlier insurance of 1000*l.*, is the whole provision I have made for my family; and what remains of the pensions after the annual payments are made, is the whole of my certain income. All beyond must be derived from my own industry. Writing for a livelihood, a livelihood is all I have gained; for, having

* Reminiscences of Samuel Taylor Coleridge, and Robert Southey. By Joseph Cottle. London. 1847. P. 424.

also something better in view, and therefore having never courted popularity, nor written for the mere sake of gain, it has not been possible for me to lay by anything. Last year, for the first time in my life, I was provided with a year's expenditure beforehand. This exposition might suffice to show how utterly unbecoming and unwise it would be to accept the rank which, so greatly to my honour, you have solicited for me, and which his majesty would so graciously have conferred; but the tone of your letter encourages me to say more.

'My life insurances have increased in value. With these, the produce of my library, my papers, and a posthumous edition of my works, there will probably be 12,000*l.* for my family at my decease. Good fortune, with great exertions on the part of my friend's surviving friends, might possibly extend this to 15,000*l.*, beyond which I do not dream of any further possibility. I had bequeathed the whole to my wife, to be divided ultimately between our four children; and having thus provided for them, no man could have been more contented with his lot, nor more thankful to that Providence on whose especial blessing he knew that he was constantly, and, as it were, immediately dependent for his daily bread.

"But the confidence which I used to feel in myself is now failing. *I was young in health and heart at my last birthday, when I completed my sixtieth year. Since then, I have been shaken at the root.* It has pleased God to visit me with the severest of all domestic afflictions, those alone excepted into which guilt enters. My wife, a true helpmate as ever man was blessed with, lost her senses a few months ago. She is now in a lunatic asylum; and broken sleep and anxious thoughts, from which there is no escape in the night season, have made me feel how more than possible it is that a sudden stroke may deprive me of those faculties, by the exercise of which this poor family has hitherto been supported. Even in the event of my death, their condition would, by our recent calamity, be materially altered for the worse; but if I were rendered helpless, all our available means would procure only a respite from actual distress.

"Under these circumstances, your letter, sir, would, in other times, have encouraged me to ask for such an increase of pension as might relieve me from anxiety on this score. Now that lay sinecures are in fact abolished, there is no other way by which a man can be served, who has no profession wherein to be promoted, and whom any official situation would take from the only employment for which the studies and the habits of forty years have qualified him. This way, I am aware, is not to be thought of, unless it were practicable as part of a plan for the encouragement of literature; but to such a plan, perhaps, these times might not be unfavourable. The length of this communication would require an apology, if its substance could have been suppressed; but on such an occasion, it seemed a duty to say what I have said; nor, indeed, should I have deserved the kindness you have expressed if I did not explicitly declare how thankful I should be to profit by it."

This letter, subscribed in the usual form, was dated Keswick, Feb. 3, 1835.

In the meantime, Southey's anxiety for the mental convalescence of his "dear Edith" daily increased; post after post was looked forward to as the messenger of hope; and as the reports of her state improved, he thus reasoned with himself:—"The far greater number," he observes, "of incurable patients in asylums are kept there to be out of the way of their respective families. This may be necessary in some cases, but where it is not necessary, it seems to me that we are no more justified in thus ridding ourselves of a painful duty, than we should be in sending a wife or a mother to die in an infirmary, that we might escape the trouble of attending either upon a death-bed." We fully appreciate the kindly feeling and force of this remark, but mental diseases are not, in respect of treatment, to be compared with bodily diseases. A person may be treated for gout or rheumatism, or any bodily ailment, as well—perhaps better—at home, than in a hospital; but this does not apply to mental disease; for if experience has ever established one fact more clearly than another, it is, that home treatment in all cases of mental disease is pernicious. When the mind is affected, the very presence of the nearest and dearest relation—probably no longer, under some existing delusion, so esteemed—provokes excitement, and the cure of the case becomes indefinitely retarded. The very case of poor Mrs. Southey will prove in the sequel an evidence against the removal of patients to their homes while they are yet imperfectly recovered, or only advancing towards convalescence. Actuated, however, by the purest and best motives, Southey determined upon removing her from the "Retreat." Accordingly he proceeded for that purpose to York, and having taken her out of the asylum, stayed with his sad charge a few days at Scarborough, whence he wrote in the following terms to his son:—

"The monotony of this week is a curious contrast to the excitement and movements of the preceding month. The first great change in your life has taken place during this interval, and I am about to enter upon not the least in mine, so different will my household be from what it has formerly been, and so much will it be reduced. Your sisters will find themselves supported in the performance of their duties; and after the emotion which our r turn must produce is over, their spirits, I doubt not, will rally. We shall always have enough to do—they as well as myself: and this certain, that they who are resigned to God's all-wise will, and endeavour to do their duty in whatever circumstances they are placed, can never be thoroughly unhappy—never, under any affliction, can find themselves without consolation and support."

This letter was dated the 27th March, 1835.

Upon returning home, he watched over his beloved patient with unremitting solicitude, and hailed with sanguine hope the slight appearance of temporary improvement; but they who know from experience how transient are the lights and shadows which pass across the deranged mind; how deceptive the lucidity which may for hours or even days seem to justify the promise of permanent restoration, will make every allowance for Southey fostering expectations which were destined never to be realized. But how did this constant watching and anxiety—this ever-recurring wear and tear of the heart and brain —operate on the mind of Southey? He was harassed not only by the lamentably mental condition of her who had been to him a true and affectionate helpmate for above forty years; but the apprehension of his children being left insufficiently provided for still depressed him. Writing to one of his friends, he tells him it never was his intention to leave his daughters to take care of their mother, thereby transferring to them a duty he was able and determined to bear; and he then adds :—

"If anything should be done for me (which is equally unwise to build upon and unjust to doubt); if, I say, my circumstances should be rendered easy, I believe it would have a happy effect upon her, who for some twenty years has been anxious overmuch on that score, though in the morning of life, when all my exertions and all her economy were required, if either had failed in their respective duties we must have sank; but her spirits then failed as little as mine."

Two days after he had written this letter—while his mind was haunted by these misgivings—he received from Sir Robert Peel the following communication :—

"My dear Sir,—I have resolved to apply the miserable pittance at the disposal of the crown on the civil pension list fund altogether to the reward and encouragement of literary exertions; and I do this on public grounds, and much more with the view of establishing a principle, than in the hope, with such limited means, of being able to confer any benefit upon those whom I shall name to the crown, worthy of the crown and commensurate with their claims. I have just had the satisfaction of annexing my name to a warrant which will add 300*l.* annually to the amount of your existing pension. You will see in the position of public affairs a sufficient reason for my having done this without delay, and without previous communication with you. I trust you can have no difficulty in sanctioning what I have done with your consent, as I have acted on your own suggestion, and granted the pension on a public principle—the recognition of literary and scientific eminence as a public claim. The other persons to whom I have addressed myself on this subject are Professor Airey, of Cambridge, the first of living mathematicians and astronomers—the first of this country at least; Mrs. Somerville, Sharon Turner, and James Montgomery, of Sheffield. Believe me, my dear sir, &c., &c.

"Dated Whitehall, April 4, 1835. "ROBERT PEEL."

This pension of 300*l.* a-year—so handsomely, and without previous communication with him, being conferred—in addition to the previous pension of 200*l.* a-year, must greatly have relieved Southey's mind from the fear of pecuniary difficulties; but how in the meantime fared it with his afflicted wife? In March, 1835, she was removed from the York Retreat, and on January the 30th, 1836, he writes to Mr. May, saying—

"There is no change in our domestic circumstances; all hope is extinguished, while anxiety remains unabated, so sudden are the transitions of this awful malady. I can never be sufficiently thankful that my means of support are no longer precarious, as they were twelve months ago. The fear of being disabled, which I never felt before, might too probably have brought on the evil which it apprehended, when my life seemed to be of more consequence to my family than at any former time, and my exertions more called for. Thank God, Sir Robert Peel set me at ease on that score. . . . . We have both great comfort in our children. Perhaps one reason why women bear affliction (as I think they generally do) better than men, is because they make no attempt to fly from the sense of it, but betake themselves patiently to the duties, however painful, which they are called upon to perform. It is the old emblem of the reed and the oak—they bend, and therefore they are not broken; and then comes peace of mind, which is the fruit of resignation. Secluded as we now are from society, my daughters find sufficient variety of employment. They transcribe a good deal for me: indeed, whatever I want extracted of any length from books—most of my notes. One room is almost fitted up with books of their binding: I call it the Cottonian Library; no patchwork quilt was ever more diversified. They have just now attired two hundred volumes in this fashion. Their pleasure indeed, in seeing the books in order, is not less that my own; and, indeed, they are the pride of my eye and the joy of my heart."

The following June he again writes, saying :—

"It is not possible for me to say when it will be fitting for me to return home. My presence, though it may be little comfort to my poor wife, is a very great one to my daughters; my spirits help to keep up theirs; and with what they have to do for me in the way of transcribing, and the arrival of letters and packets, which would cease during my absence, they would feel a great blank were they left to themselves. In her quieter moods, too, poor Edith shows a feeling towards me, the last perhaps that will be utterly extirpated."

Still there was no manifest or enduring change for the better; no appearance of the hope entertained when she was removed from the York Retreat, being realized. Three months afterwards—30th of September, 1836—he writes to Mr. Cottle :—

"How like a dream does the past appear! Through the last years of my life more than any other part! All hope of recovery, or even amendment, is over! In all reason I am convinced of this; and yet at times when Edith speaks and looks like herself, I am almost ready to look for what, if it occurred, would be a miracle. It is quite necessary that I should be weaned from this constant object of solicitude ; so far at least as to refresh myself and recruit for another period of confinement."

In the midst of this heavy affliction Southey derived support and consolation from that source whence only in periods of trouble and sickness it can be found—religion ; he entertained a strong faith in the immortality of the soul, and our recognition of each other in a future state of existence.

"I could agree with you," he observes, in a letter to one of his friends, "that personal identity unbroken by death were little to be desired if it were all—if we were to begin a new life in the nakedness of that identity. But when we carry with us in that second birth all that makes existence valuable, our hopes and aspirations, our affections, our sympathies, our capacities of happiness and of improvement; when we are to be welcomed into another sphere by those dear ones who have gone before us, and are in turn to welcome those whom we left on earth, surely of all God's blessings the revelation which renders this certain is the greatest. There have been times in my life when my heart would have been broken if this belief had not supported ; at this moment it is worth all the world could give."

While he was writing these words, his "poor Edith" was dying.

"The end," he now writes, "cannot be far off, and all is going on mercifully. For several days when I have supported her down stairs, I have thought it was for the last time ; and every night when she has been borne up, it has seemed to me that she would never be borne down alive. Thank God, there is no pain—no suffering of any kind ; and only such consciousness as is consolation."

Another pause, and we then read :—

"It pleased God to release my poor Edith this morning (Nov. 16, 1837) from a pitiable state of existence, though we have always had the consolation of thinking it was more painful to witness than to endure. She had long been wasting away, and for the last month rapidly. For ten days she was unable to leave her bed. There seemed to be no suffering till excess of weakness became pain, and at no time any distress of mind; for being sensible where she was, and with whom, and of the dutiful affection with which she was attended, she was sensible of nothing more. My poor daughters have been mercifully supported through their long trial. Now that the necessity for exertion is over, they feel that prostration which in such cases always ensues. But they have discharged their duties to the utmost, and they will have their reward. It is a blessed deliverance ! the change from life to death and death to life, inexpressibly so for her."

When thus visited by any great calamity, the mind will, under its immediate shock, frequently become paralysed ; it will then recoil upon itself, and in a state of reaction, reflection will suggest for its consolation principles of the purest and highest philosophy, such as may reconcile us to the most afflicting dispensations of divine providence; but this endures not, the self-sustaining resolution presently fails, and then the stream of grief from the over-oppressed heart will burst forth with increased force and poignancy, from the very circumstance of its having been restrained and suppressed. He who has lost by death an object to whom he has been *really* attached—in whatever connexion or relationship of life—and with whom he has been habitually and familiarly associated, may thus reason with himself, and obtain for awhile a mastery over his feelings; but the victory will be of short duration, for, despite himself, in solitary hours he will feel a gap—a void in his existence—which nothing can fill up. It is time only can soothe, if it ever can heal such wounds, and "what deep wounds ever heal without a scar?" This Southey keenly felt. During the first three years that his wife was so afflicted, we are told that he—

"Bore up wonderfully; and after the first shock had passed away, his spirits, though of course not what they had been, were uniformly cheerful; indeed, he had found in the performance of a sacred duty, that peace and comfort which in such paths is ever to be found; but it was otherwise after her death. When the necessity for exertion ceased, his spirits fell, and he became an altered man. Probably, the long-continued effort now began to tell upon him, and the loss of her who for forty years, in sickness and in health, had been the constant object of his thoughts, now caused a blank that nothing could fill up."

He himself writes :—

"This event could not have been regarded otherwise than as a deliverance at any time, since there ceased to be a hope of mental restoration; and for several weeks it was devoutly to be desired. Yet it has left a sense of bereavement which I had not expected to feel, lost as she had been to me for the last three years, and worse than lost. During more than two-thirds of my life she had been the chief object of my thoughts and I of hers. No man ever had a truer helpmate. No children a more careful mother. No family was ever more wisely ordered; no housekeeping ever conducted with greater prudence or greater comfort. Everything was left to her management, and managed so quietly, and so well, that except in times of sickness and sorrow, I had literally no cares. I always looked upon it as conducing much to our happiness that we were of the same age, for in proportion to any perceptible disparity on that point, the marriage union is less complete, and so completely was she part of myself, that the separation makes me feel like a different creature. While she was herself I had no sense of growing old, or at most only such as the mere lapse of time brought with it; there was no weight of years upon me, my heart continued young, and my spirits retained their youthful buoyancy. Now the difference of five-and-thirty years between me and Bertha continually makes me conscious of being an old man. There is no one to partake with me the recollections of the best and happiest portion of my life; and for that reason, were there no other, such recollections must henceforth be purely painful, except when I connect them with the prospect of futurity."

Again Southey returned with increased ardour to his literary pursuits; and about the beginning of the year 1839, rumours were abroad that he contemplated a second marriage. Habitually domestic, he felt the want of a companion and helpmate, and his thoughts turned towards a lady who had sympathised with him in all his afflictions, and who now consented to share with him the fortunes of his declining years—Miss Caroline Bowles, whose name is well known as an accomplished poetess. In a letter, dated February 18, 1839, he says :—

"You may possibly have heard from the newspapers that I have resolved upon a second marriage. I need not say that such a marriage must be either the wisest or the weakest action of a man's life. But I may say that in points of age, long and intimate acquaintance and conformity of opinions, principles, and likings, no persons could be better suited to each other."

In another letter he observes—

"Reduced in number as my family has been within the last few years, my spirits would hardly recover their habitual and healthful cheerfulness, if I had not prevailed on Miss Bowles to share my lot for the remainder of our lives. There is just such a disparity of age as is fitting; we have been well acquainted with each other for more than twenty years, and a more perfect conformity of disposition could not exist; so that in resolving upon what must be either the weakest or the wisest act of a sexagenarian's life, I am well assured that according to human foresight I have judged well and acted right, both for myself and my remaining daughter."

Accordingly Robert Southey was married to Miss Caroline Bowles, at Boldre church, on the 5th June, 1839. It was hoped and anticipated that this marriage would have had the effect of rallying his health and spirits ; but unhappily it proved otherwise ;—"The tree will wither long before it fall ;" and as he had pathetically said in his letter to Sir Robert Peel, he had been "shaken at the root." On his way home after his marriage (in 1839) he passed, with his wife, a few days in London, when his friends plainly perceived—that which, from the altered style of the few brief letters they had lately received from him, they already feared—that his intellectual faculties were becoming impaired. One of his most intimate friends at this period writes as follows :—

"I have just come home from a visit which affected me deeply. . . . It was to Southey, who arrived in town to-day from Hampshire, with his wife. He is, I fear, much altered. The animation and peculiar clearness of his mind quite gone, except a gleam or two now and then. What he said was much in the spirit of his former mind as far as the matter and meaning went, but the tone of strength and elasticity was wanting. The appearance was that of a placid languor, sometimes approaching to torpor, but not otherwise than cheerful. He is thin and shrunk in person, and that extraordinary face of his has no longer the fire and strength it used to have, though the singular cast of the features and the habitual expression make it still a most remarkable phenomenon. Upon the whole I came away with a troubled heart. . . He has been living since marriage in Hampshire, where he has not had the aid of his old habits and accustomed books to methodize his mind. All this considered, I think we may hope that a year or two of quiet living at his own house may restore him. The easy cheerful temperament will be greatly in his favour. You must help me to hope this, for I could not bear to think of the decay of that great mind and noble nature—at least not of its premature decay."

On the following day the same friend writes—

"I think I am a little relieved about Southey to-day. I have seen him three times in the course of the day, and on each occasion he was so easy and cheerful, that I should have said his manner and conversation did not differ, in the most part, from what it would have been in former days, if he had happened to be very tired. I say for the most part only, though; for there was once an obvious confusion of ideas. He lost himself for a moment; he was conscious of it, and an expression passed over his countenance which was exceedingly touching—an expression of pain and also resignation."

His friends now urged the necessity of diverting his mind by change of scene, and a short tour on the continent was proposed. A party of six was soon formed; and it was agreed that they should proceed through Normandy and Bretagne—visiting the principal towns—and that they should separate at Paris. The travellers accordingly set out upon their route. Southey took much interest in all he saw while actually travelling; the change and excitement seemed to keep up his spirits; still his movements were slower than usual; he was subject to frequent fits of absence; and there was an indecision in his manner and an unsteadiness in his step never before observed.

"The point," says his son, "in which he seemed to fail most was that he continually lost his way, even in the hotels we stopped at; and perceiving this I watched him constantly, as, although he himself affected to make light of it, and laughed at his own mistakes, he was evidently painfully conscious of his failing memory in this respect. His journal also, for he kept up his old habit of recording minutely all he saw, is very different from that of former journeys—breaks off abruptly when about two-thirds of our journey was completed; and shows, especially towards the close, a change in his hand-writing, which, as his malady crept on, became more and more marked, until in some of the last notes he ever wrote, the letters are formed like the early efforts of a child. . . . . Much of my father's failure, in its early stages, was at first ascribed by those anxiously watching him to repeated attacks of influenza, at that time a prevailing epidemic, from which he had suffered greatly, and to which he attributed his own feelings of weakness; but alas! the weakness he felt was as much mental as bodily—though he had certainly declined much in bodily strength—and this, after his return home, gradually increased upon him. The uncertain step, the composed manner, the eye once so keen and so intelligent, now either wandering or restlessly fixed, as it were, in blank contemplation; all showed that the overwrought mind was worn out. One of the plainest signs of this was the cessation of his accustomed labours; but while doing nothing—with him how plain a proof that nothing could be done—he would frequently anticipate a coming period of his usual industry. His mind, while any spark of his reasoning powers remained, was busy with its old day-dreams,—the history of Portugal—the history of monastic orders—all were soon to be taken in hand in earnest, all completed, and new works added to these. For a considerable time after he had ceased to compose he took pleasure in reading, and the habit continued after the power of comprehension was gone. His dearly-prized books, indeed, were a pleasure to him almost to the end, and he would walk slowly round his library looking at them, and taking them down mechanically."

We must now, as the last days of Southey draw to a close, picture to ourselves that once athletic frame, feeble and emaciated, sitting perhaps in "the Cottonian library," in which he formerly took a scholar-like yet playful delight, and gazing vacantly around him. We can conceive nothing more melancholy than the account which Wordsworth gives to Lady Bentinck of a visit to him:—

"I ought not," he says, "to forget that two days ago I went over to see Mr. Southey, or rather Mrs. Southey, for he is past taking pleasure in the presence of any of his friends. He did not recognise me till he was told. Then his eyes flashed for a moment with their accustomed brightness, but he sank into the state in which I had found him—patting with both hands his books affectionately like a child. Having in vain attempted to interest him by a few observations, I took my leave. It was for me a mournful visit, and for his wife also."

We learn also from Mr. Cottle, to whom Wordsworth communicated the particulars of this visit, that while he took books down from the shelves of his library from mechanical habit, he did not know his own children. In this state he lingered, gradually became weaker, until the 21st of March [1843], when, after a slight attack of fever, he "passed away without any outward signs of pain."

"It was a dark and stormy morning," says his biographer, "when he was borne to his last resting place, at the western end of the beautiful churchyard of Crosthwaite. There lies his dear son Herbert. There his daughters, Emma and Isabel. There Edith, his faithful helpmate of forty years. But few besides his own family and immediate neighbours followed his remains. His only intimate friend within reach, Mr. Wordsworth, crossed the hills that wild morning to be present."

